# Genomic Insights Into Early‐Stage Selective Filtering During the Transport Stage of Biological Invasions

**DOI:** 10.1111/eva.70177

**Published:** 2025-11-06

**Authors:** Yiyong Chen, Ruiying Fu, Aibin Zhan

**Affiliations:** ^1^ Research Center for Eco‐Environmental Sciences Chinese Academy of Sciences Beijing China; ^2^ College of Ecology and Environment Inner Mongolia University Hohhot China; ^3^ University of Chinese Academy of Sciences, Chinese Academy of Sciences Beijing China

**Keywords:** biological invasion, environmental filtering, functional genomics, invasive ascidian, maritime transport, salinity stress

## Abstract

Marine biological invasions, increasingly facilitated by maritime transport, represent a major dimension of global change, threatening biodiversity, ecosystem services, and human well‐being worldwide. Although the factors shaping invasion success have been widely studied, the evolutionary processes occurring during the transport stage remain poorly understood. Using high‐salinity selection experiments with the model invasive ascidian *Ciona robusta*, we tested whether transport‐related stress imposed genotype‐dependent filtering. We quantified survival dynamics and employed whole‐genome resequencing together with transcriptomic profiling to characterize genome‐wide responses to environmental filtering. Survival analyses revealed significant mortality differences among genotypes under hypersaline conditions. Whole‐genome resequencing of survivors identified genomic regions with marked genetic differentiation and allele frequency shifts, particularly in osmoregulatory genes such as solute carriers and ion channels. Transcriptomic profiling further demonstrated genotype‐specific expression patterns consistent with stress responses, highlighting the functional relevance of candidate variants. Collectively, our findings show that transport stress drives genotype‐dependent survival and functional genomic signatures consistent with selection. Acknowledging transport as an evolutionary filter and integrating such processes into invasion risk frameworks are essential for developing effective management and prevention measures in an era of accelerating global trade and climate change.

## 1 Introduction

Biological invasions are increasingly recognized as an important aspect of global change, contributing substantially to biodiversity loss and posing serious threats to ecosystem functioning and human well‐being in the Anthropocene (Diagne et al. 2021; Lopez et al. 2022; Turbelin et al. 2023). Accelerated by globalization, such as intensified human mobility and international transportation, the unintentional translocation of non‐native species across biogeographic boundaries has led to the widespread introduction of novel taxa into local ecosystems worldwide (Sardain et al. 2019; Seebens et al. 2016). Maritime shipping, which currently accounts for nearly 90% of global trade and is projected to grow significantly by 2050, plays a central role in driving marine biological invasions (Chen et al. 2025; Sardain et al. 2019). Key invasion pathways associated with shipping, such as ballast water discharge and biofouling on vessel hulls, account for the majority of marine introductions (Bailey et al. 2020). Once released, invasive species can successfully establish and spread rapidly, especially in marine ecosystems that lack physical barriers, making them difficult and costly to control or eradicate. Therefore, a deep understanding of the ecological and evolutionary mechanisms that drive invasion success is essential for advancing both theoretical research and practical management, particularly in the context of accelerating global change (Chen, Gao, et al. 2024; Gao et al. 2022; Ren et al. 2025).

Invasion success is not a single event but the cumulative result of multiple ecological and evolutionary filters acting sequentially across stages such as transport, introduction, establishment, and spread (Blackburn et al. 2011; Gioria et al. 2023). Although a majority of studies have concentrated on the post‐introduction phases, particularly establishment and spread, the transport stage remains comparatively understudied (Baños‐Villalba et al. 2021; Briski et al. 2018). This initial phase, during which organisms are passively moved across biogeographic boundaries via anthropogenic vectors such as shipping ballast water constitutes a critical yet often overlooked ecological barrier (Briski et al. 2018). Throughout transport, organisms face acute and fluctuating environmental stressors, including changes in temperature and salinity, hypoxia, and nutrient scarcity, which can impose severe physiological challenges and sharply reduce survival rates (Ghabooli et al. 2016). Crucially, only individuals that withstand these stressors progress to later invasion stages. Consequently, the transport stage can serve as a pivotal barrier that selectively shapes the composition and evolutionary potential of emerging invasive populations (Briski et al. 2018). Despite this, it is frequently regarded as a stochastic or neutral phase in invasion models, primarily influenced by propagule pressure and random mortality, resulting in its evolutionary importance being underrepresented in both conceptual and predictive frameworks.

During transport, both propagule pressure (i.e., the number of individuals introduced) and colonization pressure (i.e., the number of species introduced) have long been recognized as central predictors of invasion success (Hayes and Barry 2008; Lockwood et al. 2005; Ricciardi et al. 2017). This “numbers game” paradigm assumes that greater introduction effort increases the likelihood of establishment, often overlooking variation in individual quality, fitness, or adaptive traits (Mueller et al. 2017). However, emerging studies have challenged this view by emphasizing the role of selective filtering during the transport stage (Baños‐Villalba et al. 2021; Briski et al. 2012, 2018). Environmental stressors encountered during translocation can impose non‐random mortality—eliminating maladapted individuals while favoring those possessing pre‐existing variations (e.g., genetic, epigenetic, or phenotypic) that enhance survival (Mueller et al. 2017). Building on this, Briski et al. (2018) proposed a selection‐based transport model, framing anthropogenic vectors as both dispersal mechanisms and agents of environmental filtering. Despite these conceptual advances, empirical evidence directly linking early‐stage environmental filtering to genomic or functional adaptation remains limited, constraining our ability to predict and manage biological invasions in the context of rapidly escalating global maritime transportation.

Elucidating how natural selection reshapes population genomic composition during the transport stage requires well‐designed experiments and suitable model systems. Most existing studies rely on observational or correlative data, which often cannot distinguish stochastic mortality from selection‐driven survival (Baños‐Villalba et al. 2021; Briski et al. 2018). Furthermore, few investigations have explicitly connected physiological responses to their underlying genomic mechanisms (Marin et al. 2020; Sun et al. 2020), leaving critical gaps in our understanding of the molecular basis of rapid adaptation. While in situ studies using common transport vectors, such as ballast water, offer valuable ecological realism, they are often logistically challenging and limited in mechanistic resolution (Briski et al. 2018; Sylvester et al. 2011). In contrast, laboratory‐based selection experiments provide a powerful alternative by enabling controlled simulation of transport‐related stressors and detailed monitoring of phenotypic and genomic responses (Briski et al. 2018; Han et al. 2025). For example, McKenzie et al. (2012) demonstrated that genotype‐specific tolerance to copper‐based antifouling paint facilitated the spread of the sessile bryozoan *Watersipora subtorquata* via ship hulls, highlighting the role of environmental filtering in selecting for resistant genotypes. With the advent of high‐throughput sequencing, detecting genome‐wide signatures of selection and identifying adaptive loci linked to stress tolerance and invasion potential has become increasingly feasible (Brennan et al. 2022; Laurentino et al. 2020). In this study, we employed the model invasive ascidian *Ciona robusta* to investigate genomic responses to selection during the transport stage. *C. robusta* is a globally marine invasive species, likely native to the Northwest Pacific, and has been widely introduced into global coastal ecosystems, including those of China (Zhan et al. 2010, 2015). It is a well‐established model in developmental biology, evolutionary and ecological studies, as well as invasion science (Dehal et al. 2002; Zhan et al. 2015). It exhibits broad environmental tolerance (from −1°C to 30°C; salinity 8‰ to 40‰; Briski et al. 2013), possesses a fully annotated small genome (approximately 123 Mb, Satou et al. 2019), and is known to spread via well‐documented anthropogenic vectors such as ballast water, aquaculture, and hull fouling (Zhan et al. 2010). These attributes make it an ideal system for exploring the molecular and evolutionary mechanisms underlying environmental filtering during the transport stage of biological invasions.

**FIGURE 1:**
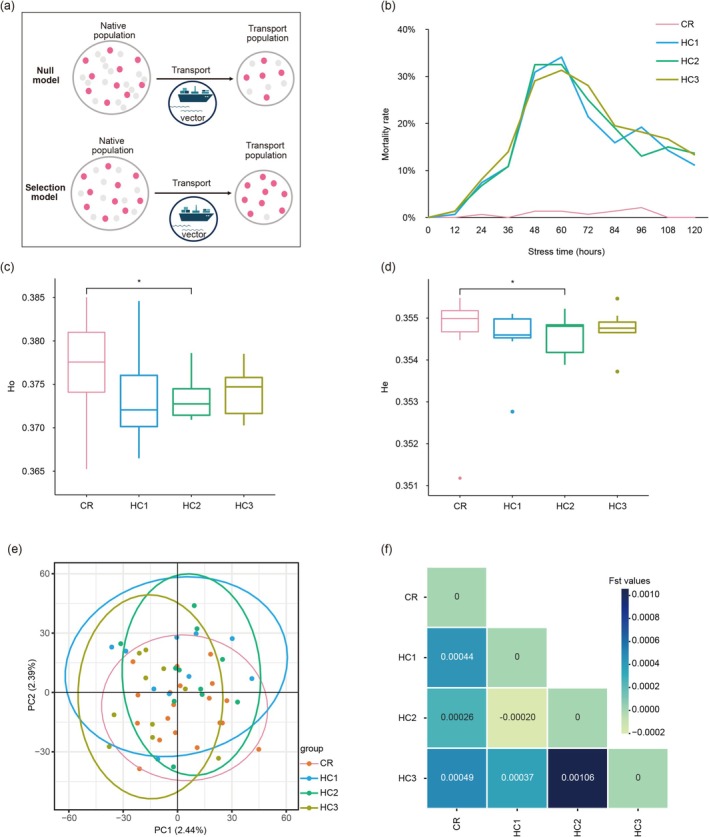
Selection during the transport stage influences mortality and genome‐level diversity. (a) Conceptual framework illustrating the selection model during the transport stage of marine invasions. Small circles represent individual genotypes, with red color indicating higher fitness under transport conditions. Under the selection model, environmental filtering alters genotype composition during transport. (b) Mortality under high‐salinity stress (40‰) across three treatment replicates (HC1–HC3) and a control group (CR, 30‰). (c, d) Boxplots comparing observed (*H*_o_) and expected (*H*_e_) heterozygosity across groups. (e) Principal component analysis (PCA) based on genomic SNPs. (f) Heatmap of pairwise *F*_ST_ values among treatment and control groups. The asterisk (*) denotes statistical significance at a False Discovery Rate (FDR)–adjusted *p* < 0.05.

While transport exposes organisms to multiple, fluctuating stressors, we focused on salinity, a dominant and rapidly varying environmental factor in major marine invasion vectors, including both hull‐fouling and ballast water. Salinity shifts can exceed 15‰ during shipping‐mediated range expansions of invasive ascidians including *Ciona* (Briski et al. 2013). Given the species' osmoregulatory physiology and the experimental tractability of salinity, we targeted it as the primary driver of transport‐stage selective filtering. In this study, we conducted high‐salinity selection experiments under controlled laboratory conditions to simulate environmental stress associated with transport and assess genome‐wide responses to environmental filtering. We tracked survival dynamics, performed whole‐genome resequencing to detect changes in genetic diversity and allele frequencies, and identified candidate loci, genes, and pathways potentially under selection. Complementary transcriptomic profiling was employed to examine differential gene expression and investigate the functional relevance of key candidate adaptive genes under salinity stress. Together, these integrated approaches provide experimental evidence that transport‐relevant stress can reshape the genomic architecture of invasive populations prior to their introduction (Figure 1a). By linking ecological filtering to genomic and transcriptional responses, our study offers novel insights into the evolutionary dynamics during the transport stage of biological invasions, further supporting the development of more predictive, mechanism‐based frameworks for managing biological invasions under accelerating global change.

## 2 Materials and Methods

### 2.1 Sample Collection and High‐Salinity Selection Experiments

We used an invasive‐range population from China because our study focuses on within‐population, genotype‐dependent filtering during the transport stage. Adult *C. robusta* individuals were collected from an aquaculture facility in Dalian, Liaoning Province, China (38°49′19′′ N, 121°2′28′′ E), and transported immediately to the laboratory. Species identification was confirmed using both morphological and molecular methods to distinguish *C. robusta* from its sympatric congener *C. savignyi* (Smith et al. 2012). Specimens were acclimated for 1 week under ambient seawater conditions (salinity 30‰; temperature 22°C), after which healthy individuals were selected for salinity stress experiments. To simulate environmental filtering during the transport stage, a high‐salinity treatment (40‰) was selected based on the assessments of salinity tolerance in *C. robusta* (Yan et al. 2025) and documented introductions into hypersaline habitats such as the Red Sea, where surface seawater salinity commonly exceeds 40‰ (Chen et al. 2018). The experiment consisted of one control group (30‰; CR) and three replicate high‐salinity treatment groups (40‰; HC1–HC3). Each treatment replicate was maintained in an independent aerated tank (200 individuals per tank). We used a 5‐day window to capture acute survival sorting relevant to short port‐to‐port segments and tank‐holding cycles, and to remain within established salinity‐tolerance assay windows for ascidians. Mortality was monitored at a 12‐h interval over a 5‐day exposure period. At each 12‐h census, we recorded the number of dead individuals per tank and calculated interval mortality as: Interval mortality (%) = (Deaths during the interval/Number alive at the start of the interval) × 100. Mortality distributions between control and treatment groups were compared using the two‐sided Kolmogorov–Smirnov (K–S) test. The siphon was selected as it is directly exposed to external seawater and plays a key role in osmoregulation, making it an ideal tissue for detecting responses to osmotic stress (Chen, Ni, et al. 2024). Survivors were collected at the end of the experiment, and siphon tissues were dissected, flash‐frozen in liquid nitrogen, and stored at −80°C for subsequent genomic and transcriptomic studies.

### 2.2 Whole‐Genome Resequencing and Genomic Diversity Analysis

Genomic DNA was extracted using the DNeasy Blood and Tissue Kit (QIAGEN; Cat. No. 69506) and quantified with a NanoDrop 2000 spectrophotometer (Thermo Fisher Scientific, USA). Paired‐end sequencing targeting approximately 30× coverage was performed on an Illumina HiSeq 4000 platform. Raw sequencing reads were quality‐ and adapter‐trimmed using Trimmomatic v0.36 (Bolger et al. 2014). Clean reads were aligned to the *C. robusta* reference genome (HT version; http://ghost.zool.kyoto‐u.ac.jp/download_ht.html) using BWA‐MEM v0.7.12 (Li and Durbin 2009). Sorted alignments were processed with SAMtools v1.979 (Li et al. 2009), and PCR duplicates were removed using the MarkDuplicates function in Picard v2.18.11 (http://broadinstitute.github.io/picard/). Single nucleotide polymorphism (SNP) calling was performed using GATK HaplotypeCaller v4.2.3.0 (McKenna et al. 2010), and variants were filtered based on the following criteria: QD < 2.0, FS > 60.0, ReadPosRankSum < −8.0, and QUAL < 30.0. Further filtering with VCFtools v0.1.16 (Danecek et al. 2011) retained biallelic SNPs with minor allele frequency (MAF) ≥ 0.05, missing data rate ≤ 30%, genotype quality > 10, and read depth between 10 and 100. Linkage disequilibrium pruning was conducted using PLINK v1.90 (Purcell et al. 2007) with the parameters “indep‐pairwise 50 10 0.2” (Sang et al. 2022). After these stringent filtering steps, a total of 216,557 high‐quality SNPs were retained for downstream analyses.

We calculated multiple genetic diversity metrics to compare control and treatment groups. The observed heterozygosity (*H*_o_) and expected heterozygosity (*H*_e_) were estimated for each sample using PLINK v1.90. Differences in heterozygosity between groups were assessed via Wilcoxon rank‐sum tests implemented in the R stats package. Nucleotide diversity (*P*_*i*_) was calculated in 5‐kb sliding windows using VCFtools and subsequently averaged across all windows. Allelic richness (*A*ᵣ) was estimated using the hierfstat R package (Goudet 2005). Principal component analysis (PCA) was conducted with the glPca function in the adegenet R package (Jombart et al. 2008). Pairwise genetic differentiation (Weir and Cockerham's *F*_ST_) was computed between each treatment replicate (HC1–HC3) and the control group (CR) with 1000 bootstrap replicates using the hierfstat R package.

### 2.3 Genome‐Wide Selection Signals Detection

Natural selection typically increases genetic divergence among populations and alters allele frequency distributions relative to neutral expectations (Dogantzis et al. 2024). To detect such signals, we calculated a Composite Selection Score (CSS) that integrates two complementary metrics: *F*_ST_ (genetic differentiation) and *P*_*i*_ (nucleotide diversity) (Dogantzis et al. 2024). Specifically, *F*_ST_ quantifies genetic differentiation between groups, while *P*_*i*_ reveals selective sweeps through localized reductions in nucleotide diversity. Pairwise *F*_ST_ and *P*_*i*_ values were estimated in 5‐kb sliding windows using VCFtools, and *F*_ST_ values were standardized by transformation into *Z*‐scores (Dogantzis et al. 2024). Volcano plots were constructed by plotting *Z*‐transformed *F*_ST_ values (*ZF*_ST_, *y*‐axis) against the log_2_‐transformed ratio of nucleotide diversity between groups (*x*‐axis; log_2_[*P*_*i*__CR/*P*_*i*__HC]). Candidate selection outlier intervals were defined as those falling within the top 5% of *ZF*_ST_ and exhibiting positive *P*_*i*_ ratios.

To establish null expectations, allele frequency changes were compared to fourfold degenerate (4DTV) sites assumed to be neutral. Candidate SNPs were required to meet two criteria: (1) *F*_ST_ values above the 95th percentile of the empirical distribution, and (2) allele frequency shifts exceeding the 95th percentile of the neutral 4DTV distribution. This dual‐criteria approach reduces the likelihood of false positives expected under neutrality. Under the hypothesis that loci subject to selection exhibit pronounced allele frequency shifts between control and treatment groups, whereas neutral loci—assumed to be influenced primarily by genetic drift—show minimal changes (Buffalo and Coop 2020), we compared allele frequency differences at *F*_ST_ outlier SNPs within CSS intervals to those at neutral sites. Putatively neutral loci were defined as 4DTV identified using SNPeff v4.3t (Cingolani et al. 2012) and located outside CSS intervals. Major allele frequency differences were calculated using VCFtools, and SNPs exhibiting allele frequency changes exceeding the 95th percentile or falling below the 5th percentile of the 4DTV distribution were considered significant (Dogantzis et al. 2024). SNPs meeting both criteria—*F*_ST_ outliers with significant allele frequency shifts—were designated as focal SNPs within CSS intervals.

### 2.4 Functional Annotation and Enrichment Analysis

Focal SNPs were annotated using SNPeff v4.3t to determine their chromosomal location, genomic region (e.g., intronic, exonic, and untranslated regions), and predicted functional effects, which were classified as synonymous or non‐synonymous substitutions. Gene ontology (GO) enrichment analysis was performed with the clusterProfiler R package (Xu et al. 2024), applying a false discovery rate (FDR) threshold of adjusted *p* < 0.05 for significance. Enriched GO terms were visualized using the dotplot function in the ggplot2 R package (http://ggplot2.tidyverse.org). To identify salinity‐specific functional categories, we focused on GO terms containing keywords such as “ion,” “channel,” “transport,” “water,” “chloride,” “potassium,” “homeostasis,” “urine,” “ATP (adenosine 5′‐triphosphate),” and “metabolic” (Heckwolf et al. 2020). Additionally, Kyoto Encyclopedia of Genes and Genomes (KEGG) pathway enrichment analysis was conducted using the enricher function in clusterProfiler, with significance defined at FDR < 0.05.

### 2.5 Transcriptomic Analysis

For RNA‐seq, total RNA was extracted from the siphon tissue of each surviving individual in the control and treatment groups using TRIzol reagent (Invitrogen, USA) following the manufacturer's instructions. RNA quality and quantity were assessed using a NanoDrop 2000 spectrophotometer (Thermo Fisher Scientific, USA), and integrity was verified by agarose gel electrophoresis and an Agilent 2100 Bioanalyzer (Agilent Technologies, USA). High‐quality RNA samples were used for first‐strand cDNA synthesis (PrimeScript II; TaKaRa) and subsequent RNA‐seq library preparation using the NEBNext Ultra RNA Library Prep Kit (Illumina‐compatible). Libraries were sequenced (2 × 150 bp) on the Illumina HiSeq Xten platform. Raw reads underwent quality control with FastQC v0.11.9 and trimming with Trimmomatic v0.39. Clean reads were aligned to the reference genome using Hisat2 v2.2.1 (Kim et al. 2019). Gene‐level read counts were obtained using featureCounts v2.0.1 (Liao et al. 2014) and normalized to transcripts per million (TPM). Differential gene expression analysis between treatment and control groups was performed with DESeq2 in R (Love et al. 2014). Genes exhibiting fold change > 2 and Benjamini–Hochberg adjusted *p* < 0.05 were considered significantly differentially expressed. To evaluate whether the observed differential expression could arise by chance under our single‐population, unbalanced design, we performed a label‐permutation test with 1000 iterations. Volcano plots were generated using ggplot2, and expression differences of focal genes were statistically assessed via Wilcoxon rank‐sum tests.

## 3 Results

### 3.1 Mortality and Genome‐Level Diversity Changes Under High‐Salinity Stress

To test whether salinity stress imposes selective mortality, we compared survival outcomes between a control group (CR; 30‰ salinity) and three replicate high‐salinity treatments (HC1–HC3; 40‰). Mortality trajectories and genomic variation of survivors were then analyzed. After stress, all replicates exhibited bell‐shaped mortality curves, characterized by peak mortality between 48 and 72 h post‐exposure (Figure 1b). Mortality rates were significantly elevated in the high‐salinity treatment groups compared to the control group (CR) (two‐sided Kolmogorov–Smirnov test, *D* = 0.8182, *p* = 0.0004). Mortality rates decelerated and plateaued during the later exposure phase, indicating the selective survival of a subset of individuals with enhanced tolerance to salinity stress (Figure 1b).

We sequenced the genomes of 50 surviving individuals, comprising 19 from the control group and 31 from the high‐salinity treatments (HC1: *n* = 10; HC2: *n* = 11; HC3: *n* = 10). After stringent quality filtering, 216,557 high‐quality SNPs were retained for downstream analyses. Compared to the control, treatment groups exhibited a reduction in genome‐wide genetic diversity, most notably in observed and expected heterozygosity (*H*_o_ and *H*_e_) within HC2 (Wilcoxon rank‐sum test; FDR‐adjusted *p* = 0.0370 for *H*_o_ and FDR‐adjusted *p* = 0.0260 for *H*_e_; Figure 1c,d; Table S1). PCA showed that all samples clustered closely, yet within‐group genetic variation increased in the treatment groups relative to the control (Figure 1e). Pairwise *F*_ST_ values between control survivors (CR) and each high‐salinity treatment replicate (HC1–HC3) were low and not statistically significant (mean *F*_ST_ = 0.0004, *p* > 0.05), indicating that mortality induced by high salinity treatment did not lead to genome‐wide genetic differentiation (Figure 1f).

### 3.2 Genomic Variation Consistent With Signals of Selection Under High‐Salinity Stress

**FIGURE 2:**
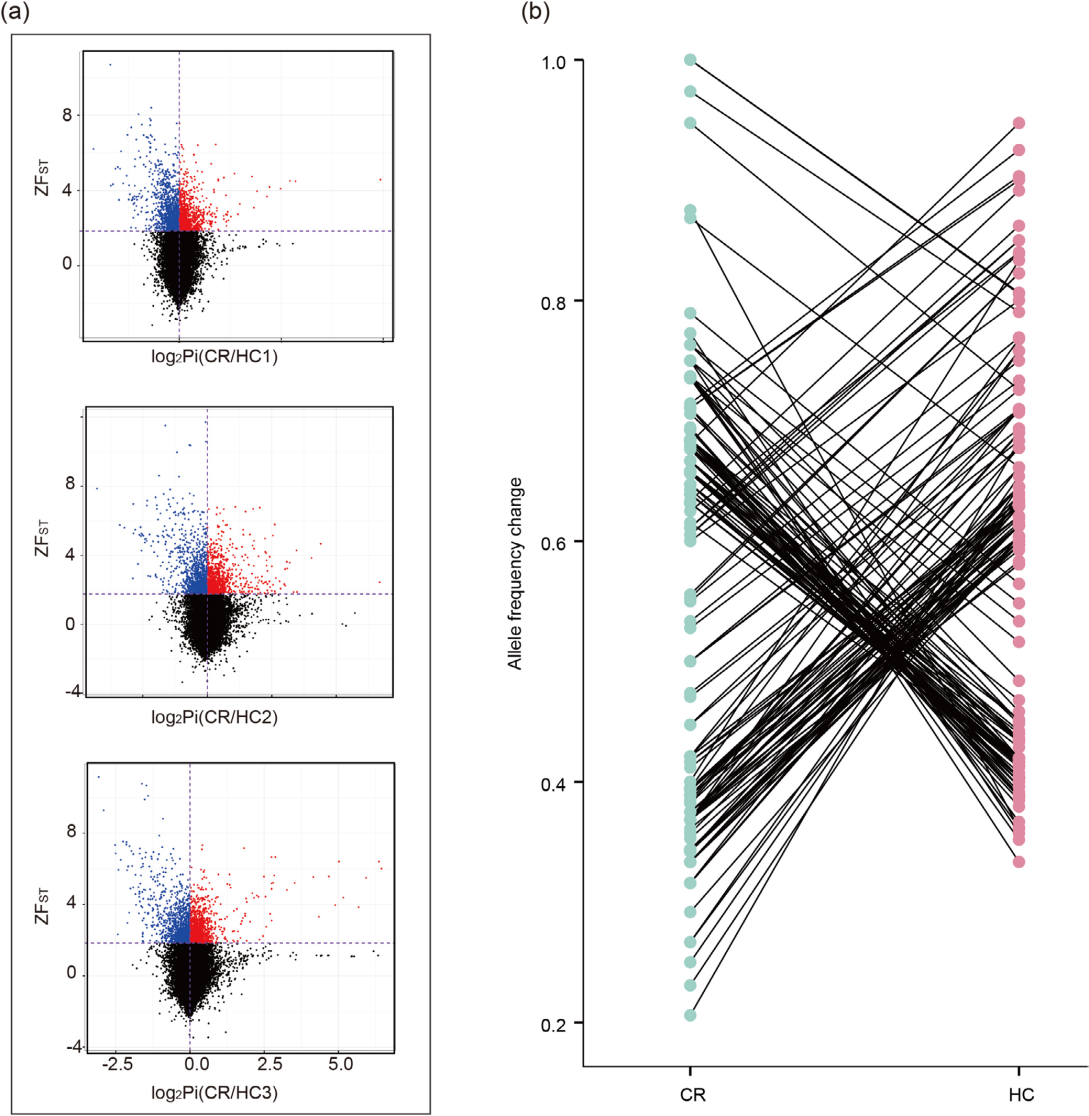
Genome‐wide signatures of selection under high‐salinity stress. (a) Volcano plots of *Z*‐transformed *F*_ST_ and log_2_‐transformed nucleotide diversity (*P*_*i*_) ratios for three replicate high‐salinity treatments. Red and blue dots indicate outlier intervals under selection in the high‐salinity and control groups, respectively, which were both defined as those exceeding the 95th percentile for *ZF*_ST_ and showing a positive *P*_*i*_‐ratio. (b) Allele frequency changes for candidate focal SNPs between control (CR) and high‐salinity (HC) groups. SNPs meeting both criteria—*F*_ST_ outliers with significant allele frequency shifts—were designated as focal SNPs within CSS intervals.

To identify genomic regions potentially under selection, we calculated CSS that integrates *F*_ST_ and nucleotide diversity (*P*_*i*_), aiming to detect regions exhibiting high genetic differentiation alongside reduced diversity. Candidate intervals were defined as the top 5% *ZF*_ST_ windows with positive *P*_*i*_ ratios, signals consistent with selection rather than random variation (see Section 2). This analysis identified 1022, 1069, and 1149 candidate intervals in HC1–HC3 comparisons, respectively (Figure 2a). Among these, 210 intervals were shared by at least two treatment groups and were thus considered robust outlier regions indicative of selection. Within these regions, we detected 10,717 SNPs in the top 5% of *F*_ST_ values between treatment and control groups, of which 198 overlapped with CSS‐defined outlier intervals. We further assessed allele frequency shifts relative to fourfold degenerate (4DTV) sites, which serve as putatively neutral loci. As expected under genetic drift, 4DTV SNPs displayed a narrow, symmetric distribution of allele frequency changes centered near zero (Figure S1). In contrast, *F*_ST_ outlier SNPs exhibited a broader, bimodal distribution with significantly greater allele frequency shifts (Wilcoxon rank‐sum test, *p* < 0.0001; Figure S1). Using the empirical 5th and 95th percentiles of allele frequency change in the 4DTV distribution as thresholds, we identified 171 SNPs showing both high *F*_ST_ and extreme allele frequency changes, hereafter referred to as focal SNPs (Figure 2b).

### 3.3 Functional Annotation of Focal SNPs

**FIGURE 3:**
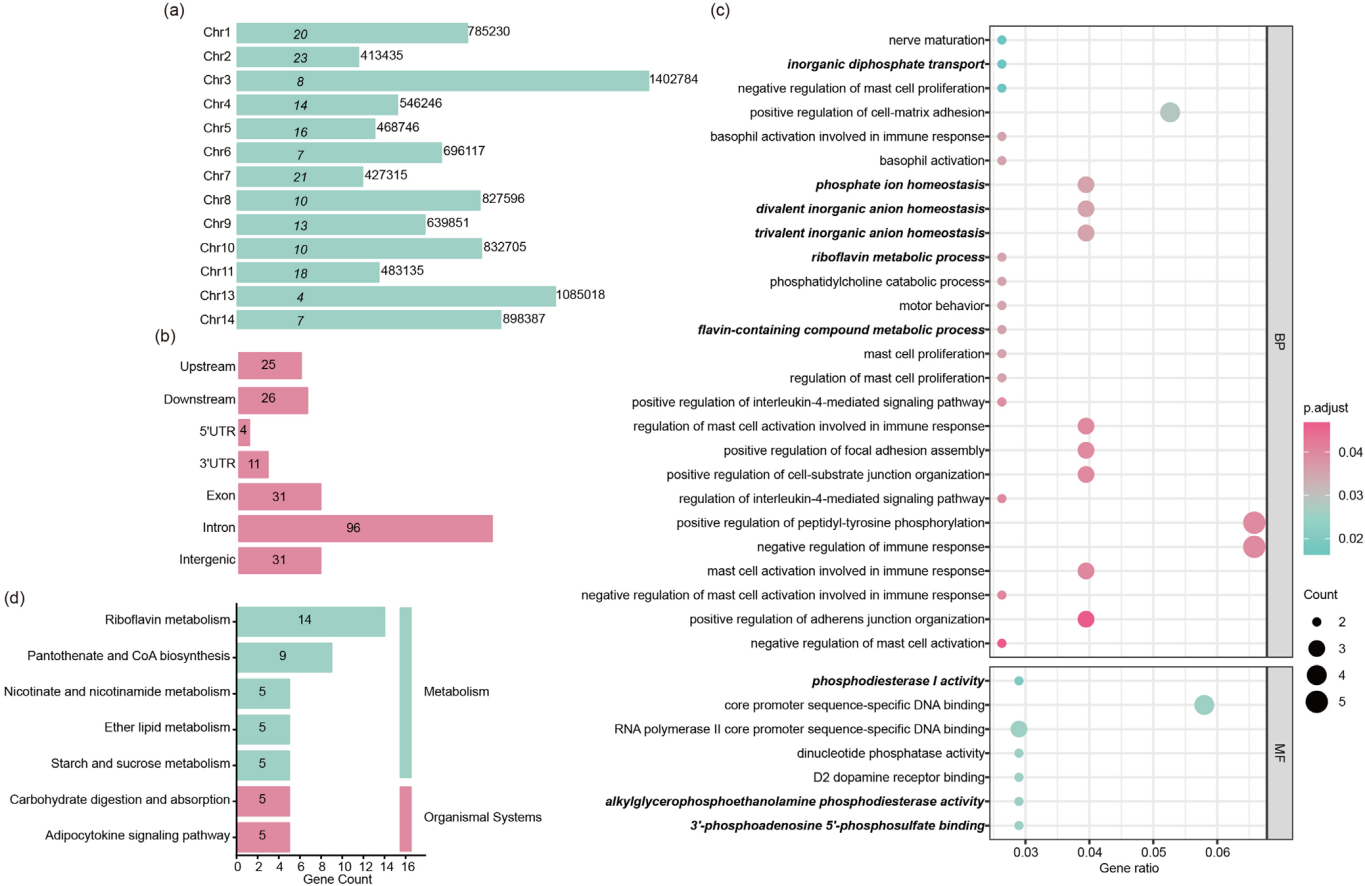
Functional annotation of candidate SNPs with selection signals. (a) Chromosomal distribution of focal SNPs and their average inter‐SNP distance. The numbers on bars indicate per‐chromosome focal‐SNP counts; the numbers on the right of bars indicate the average distance between focal SNPs on each chromosome. (b) Genomic location of focal SNPs across features such as exons, introns, upstream/downstream regions, and untranslated regions (UTRs). (c) Gene Ontology (GO) enrichment analysis of candidate genes harboring focal SNPs. GO terms potentially associated with osmoregulatory are marked with bold italics according to Heckwolf et al. (2020). (d) KEGG enrichment analysis of candidate genes associated with focal SNPs.

To infer the potential biological significance of focal SNPs, we annotated their chromosomal locations, genomic contexts, and functional categories. Focal SNPs were unevenly distributed across the genome, with chromosome 2 exhibiting the highest SNP density (average inter‐SNP distance = 413,435 bp; Figure 3a). The majority of focal SNPs were located in intronic (*n* = 96), exonic (*n* = 31), or intergenic regions (*n* = 31) (Figure 3b). Among these, 99 variants were mapped to functionally relevant regions, including 25 in promoter regions, 15 in untranslated regions (UTRs), and 6 non‐synonymous coding variants (Table S2). Annotation of 171 focal SNPs revealed 116 associated protein‐coding genes (with few genes containing more than one SNP), several of which are implicated in ion transport and osmoregulation. Notably, we identified four solute carrier (*SLC*) genes—*SLC5A8*, *SLC2A1*, *SLC2A9*, and *SLC26A5*—as well as genes encoding the potassium channel *KCNN4* and the ATP‐binding cassette transporter *ABCA5* (Table S2). For example, a synonymous SNP on chromosome 14 was mapped to *SLC5A8*, a sodium‐coupled transporter involved in salt regulation. *KCNN4* harbored a focal SNP in its upstream regulatory region and encodes a calcium‐activated potassium channel that modulates membrane potential and cellular ion flux. *ABCA5*, containing a synonymous variant, facilitates ATP‐dependent transmembrane transport of diverse substrates.

GO analysis identified 33 significantly enriched terms, encompassing fundamental cellular processes such as DNA binding, immune response, and cell adhesion, as well as metabolic pathways including riboflavin metabolism (GO:0006771) (Figure 3c; Table S3). Importantly, several GO terms related to osmoregulatory functions were enriched, including ion transport (GO:0030505 inorganic diphosphate transport), ion homeostasis (GO:0072505 divalent inorganic anion homeostasis), and ATP hydrolysis (GO:0004528 phosphodiesterase I activity). KEGG pathway analysis revealed seven significantly enriched pathways, most of which (5/7) belonged to the metabolism category, including pantothenate and CoA biosynthesis, nicotinate and nicotinamide metabolism, and ether lipid metabolism (Figure 3d).

### 3.4 Gene Expression

**FIGURE 4:**
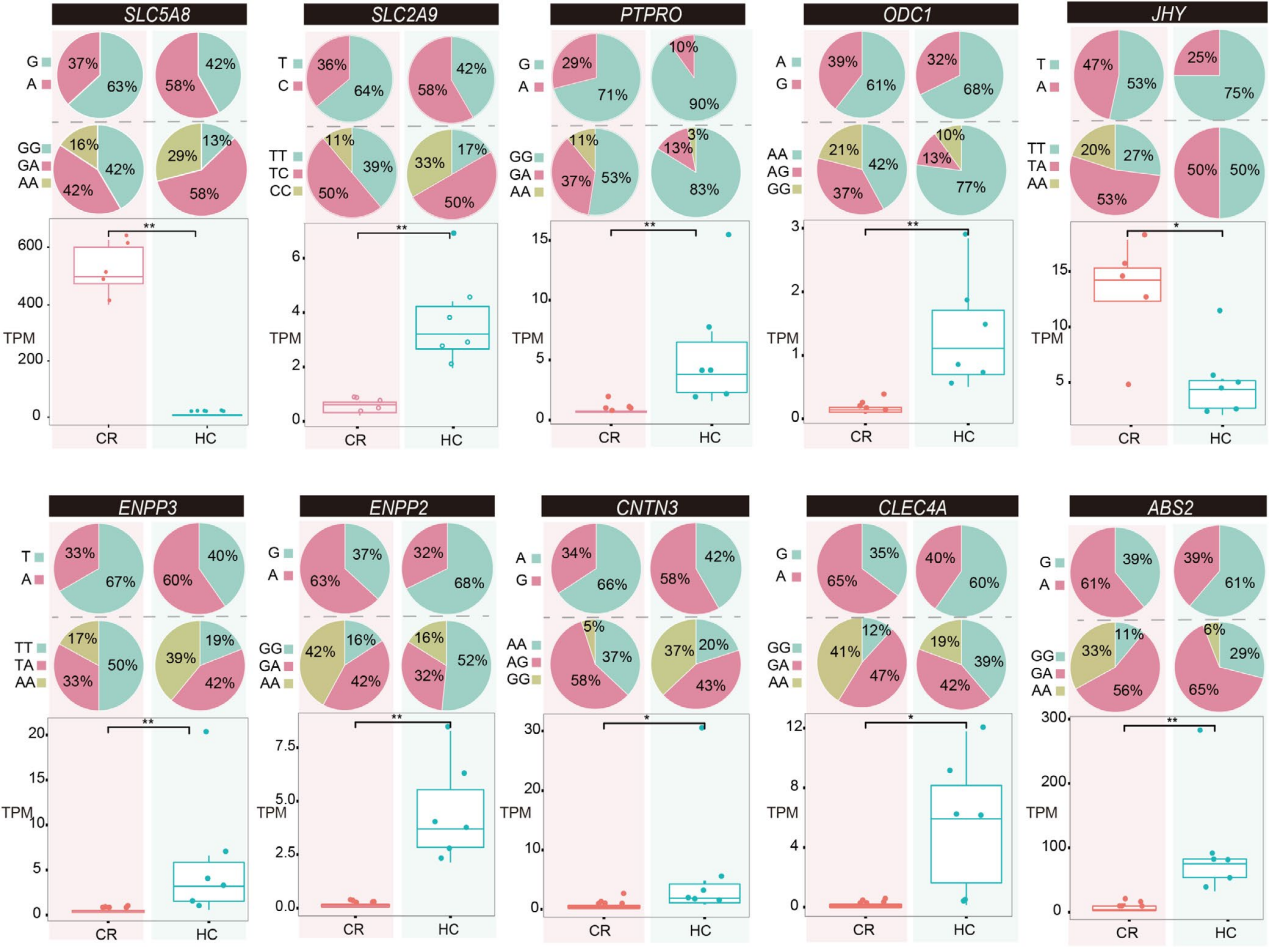
Differential expression of candidate genes potentially under selection. Ten functional genes containing focal SNPs exhibited significant differential expression between high‐salinity (HC) treatments and control (CR) groups. Changes in allele frequencies, genotype frequencies, and gene expression profiles indicate potential functional relevance under transport‐related environmental stress.

To investigate the functional relevance of potentially selected genomic regions, we performed transcriptomic profiling using RNA‐Seq. A total of 1352 genes were differentially expressed (DEGs) between control and treatment groups (fold change > 2, FDR‐adjusted *p* < 0.05), comprising 530 upregulated and 822 downregulated transcripts (Figure S2). The permutation analysis showed that the observed number of DEGs was greater than expected under the permutation null (empirical *p* < 0.05). Among the 116 genes harboring focal SNPs, 10 exhibited significant differential expression: *SLC5A8*, *SLC2A9*, *PTPRO*, *ODC1*, *JHY*, *ENPP2*, *ENPP3*, *CNTN3*, *CLEC4A*, and *ABS2* (Figure 4). Of these, eight genes were upregulated, while two were downregulated under high salinity stress. For instance, *SLC5A8* expression was markedly reduced in the treatment group (median TPM = 6.78) compared to the control (median TPM = 522.47; *p* < 0.01), correlating with a decline in the G allele frequency (control = 63%; treatment = 42%) and a reduction in the GG genotype frequency (control = 42%; treatment = 13%). Conversely, *SLC2A9* expression increased in treated individuals (median TPM = 3.58), accompanied by elevated frequencies of the C allele (58% vs. 36% in controls) and the CC genotype (33% vs. 11%). For *JHY*, the A allele frequency dropped from 47% to 25%, with complete loss of the AA genotype under high salinity, which paralleled a significant decrease in expression (median TPM = 4.91 vs. 12.82 in controls; *p* < 0.01). These genotype‐dependent differences indicate functional correlation between allele frequency shifts and gene expression under high‐salinity stress.

## 4 Discussion

The transport stage of biological invasions has traditionally been viewed through a demographic lens, with emphasis on propagule pressure and colonization pressure as primary determinants of invasion success (Briski et al. 2012; Lockwood et al. 2009). Although substantial progress has been made in understanding the ecological dynamics during the establishment stage, the role of pre‐introduction selection, particularly during the transport phase, remains poorly understood at the molecular level (Briski et al. 2018). In this study, we provide direct evidence of genotype‐dependent filtering during the transport stage, a phase underrepresented in invasion biology frameworks. Using high‐salinity selection experiments with the model invasive ascidian *C. robusta* , we show that exposure to transport‐relevant stress leads to non‐random mortality when considering the genetic diversity at the genome level. Survival curves differed significantly between the high‐salinity treatments and control (two‐sided K–S test, *p* < 0.01), indicating that certain genotypes had higher mortality under stress. We infer selection because mortality was non‐random, but an ideal test of adaptive evolution would require measuring tolerance in the offspring of survivors. Interestingly, we did not observe a significant reduction in population‐level genetic diversity, except in one stressed replicate, but we observed detectable signals of selection at functionally relevant genomic regions. Notably, we observed coordinated shifts in both genotypes and gene expression in candidate osmoregulatory genes. Together, these findings challenge the prevailing view that transport‐related mortality is purely stochastic, and instead suggest that the transport phase can act as a critical evolutionary enhancer, reshaping the genetic trajectory of invasive populations and potentially favoring stress‐tolerant genotypes for later stages.

Our findings provide empirical support for the “selection during transport” hypothesis (Briski et al. 2018), demonstrating that anthropogenic vectors such as ballast water can impose selective pressures that disproportionately favor stress‐tolerant genotypes. The transport stage represents a selective context that differs fundamentally from the establishment stage, which typically occurs over longer timescales and integrates biotic interactions as well as habitat seasonality (Blackburn et al. 2020, 2011). In contrast, conditions during the transport stage inside vectors such as ballast tanks or fouling communities are acute, episodic, and artificial, often imposing strong selection over hours to days on small, transient cohorts with limited effective population sizes (Baños‐Villalba et al. 2021; Briski et al. 2018). By pre‐selecting stress‐tolerant genotypes, transport‐stage selection can enhance survival upon release, reduce demographic stochasticity at arrival, and bias the initial genetic substrate for subsequent adaptation, thereby exerting a disproportionate influence on the invasion process despite its short duration. Consistent with this view, our 5‐day assays reveal selection‐consistent allele‐frequency shifts among survivors, particularly at functionally relevant loci involved in ion transport and osmoregulation. These results suggest that early selection acts in a spatially localized and functionally targeted manner, rather than inducing broad genome‐wide divergence. Similar patterns of localized adaptive divergence have been observed across diverse taxa (Calla et al. 2025; Sandoval‐Castillo et al. 2018; Méndez‐Cea et al. 2023), where evidence of selection at ecologically important loci/genes occurs despite minimal neutral genetic differentiation. For instance, the Northern krill *Meganyctiphanes norvegica* exhibits limited population structure yet shows evidence for selection at loci associated with photoreception and thermal tolerance (Unneberg et al. 2024). These parallels highlight the importance of focusing on functional genomic variation when evaluating early‐stage adaptive responses to environmental stressors.

Transcriptomic analyses provided insights into the potential functional significance of the candidate SNPs that emerged under stress. Survivors with novel genotypes in key genes exhibited distinct gene expression patterns. Such expression adjustments may contribute to physiological resilience under stress, consistent with a stress response role of these genes. Among the 10 genes showing significant expression changes under high‐salinity conditions, they play critical roles in physiological processes such as cellular ion homeostasis, membrane potential regulation, and ATP‐dependent transport, functions essential for maintaining osmoregulatory performance in the context of fluctuating salinity. For example, *SLC5A8* exhibited a reduced frequency of the G allele accompanied by downregulated transcript levels, whereas *SLC2A9* showed an increased frequency of the C allele alongside elevated expression. These patterns indicate genotype‐dependent gene regulation and provide mechanistic evidence of rapid physiological adaptation (Guo et al. 2025; Wang et al. 2022). Similar genotype‐expression interactions have been reported in corals and mussels, where even synonymous mutations can influence expression and codon usage, suggesting potential biological relevance (Kirk et al. 2018; Tan et al. 2023).

Compared to traditional invasion models that prioritize quantity‐based metrics such as propagule and colonization pressures, our study highlights the evolutionary significance of individual quality, specifically, the genomic architecture underlying environmental resilience. Although trait‐based invasion syndromes have traditionally emphasized life‐history characteristics and morphological plasticity as key factors driving invasive success (Blackburn et al. 2011; Ricciardi et al. 2017), the role of genomic variation within populations remains largely underexplored. As demonstrated in this study, environmental stress‐filtered genetic variants can profoundly influence adaptive potential and invasion dynamics, yet they have received comparatively little attention in invasion biology research. Thus, a functional‐genomic framework enables the identification of specific loci, genes, and pathways under selection, providing predictive insight into which genotypes are most likely to survive environmental filtering during transport. High‐salinity stress encountered during translocation may therefore act as a selective filter, disproportionately removing susceptible individuals and enriching for genotypes with enhanced physiological resilience. Consequently, the survivor pool represents a functionally non‐random subset of the source population, carrying alleles that confer fitness advantages in similarly stressful environments.

Our findings indicate that transport‐stage filtering can enrich stress‐tolerant genotypes, and this concept of functional‐genomic framework aligns with stress memory and evolutionary priming, where prior exposure to environmental challenges enhances subsequent tolerance (Yan et al. 2025; Han et al. 2025). While previous studies have primarily examined these phenomena at interspecific or community levels (Xu et al. 2025; Low‐Décarie et al. 2015), our study extends this framework to the individual and genomic scales. Specifically, we observed allele‐frequency shifts among survivors at functionally relevant loci, a pattern consistent with genotype‐dependent ecological filtering under acute salinity stress. These insights are particularly relevant for invasive species, which frequently experience genetic bottlenecks (Dogantzis et al. 2024). Selection acting on standing genetic variation during transport may help offset founder effects, as observed in other systems (Chaturvedi et al. 2021; Dogantzis et al. 2024). For instance, a recent study of *Apis cerana* invasions in Australia showed that despite severe founder events likely stemming from a single colony, selection primarily targeted standing genetic variation from the source population (Dogantzis et al. 2024). While strong environmental filtering can reduce overall genetic diversity, it may simultaneously enhance adaptive potential by purging deleterious alleles and reducing genetic load (Jiang et al. 2024, 2025). Empirical evidence from *Arabidopsis thaliana* shows that populations experiencing stronger purifying selection carry a reduced genetic load and display higher fitness proxies, such as increased fruit production, suggesting that filtering may facilitate adaptation to future climatic conditions (Jiang et al. 2025). In the context of climate change and increasing anthropogenic disturbances, early‐stage environmental filtering during transport may thus pre‐condition invasive populations for rapid adaptation, promoting range expansion and invasion success in novel and variable environments.

From a theoretical perspective, our findings challenge conventional views by indicating that evolutionary processes can initiate much earlier in the invasion continuum than previously recognized. Traditionally, the transport stage of biological invasions has been regarded as a largely neutral or random bottleneck; however, our results suggest that it functions instead as a strong selective sieve. This selective filtering can reshape the genetic composition of transported populations, thereby potentially enhancing or constraining their invasive potential even before they reach new environments (Briski et al. 2018). Recognizing the transport phase as an active evolutionary enhancer fundamentally alters our understanding of invasion dynamics and underscores the need to integrate this concept into existing invasion models. By doing so, we can improve predictions regarding the likelihood of establishment, as well as forecast adaptive trajectories of invasive populations under ongoing and future global environmental changes. From an applied management standpoint, these insights carry significant implications for biosecurity strategies and invasive species control. If selection during transport consistently enriches for genotypes tolerant to abiotic stresses such as salinity, temperature extremes, or desiccation, then manipulating these abiotic factors during transport could serve as an innovative and proactive approach to reducing the viability of high‐risk propagules before introduction.

However, several open questions remain to be addressed. While our experiment here intentionally employed a single‐factor, two‐level salinity regime to maximize power for detecting short‐window selection signals that mimic transport conditions, we explicitly acknowledge that other stressors and their potential interactions warrant investigation in future factorial designs, as common physiological or molecular pathways may be shared across different stress types (Choudhury et al. 2017; Pandey et al. 2015; Priya et al. 2023). To quantify interaction effects and selection‐gradient functions, future studies will implement factorial designs (e.g., salinity × temperature, salinity × hypoxia) with finer salinity gradients, coupled with multi‐generational assays and functional validation experiments (e.g., CRISPR gene editing, physiological assays) to test adaptive outcomes. Such approaches will help determine whether the genotype‐dependent filtering observed here translates into heritable adaptation. Moreover, the magnitude and architecture of genotype‐dependent filtering may vary with background genomic composition, demographic history, and local acclimatization, meaning that effect sizes and focal alleles could differ among populations (Chen et al. 2021; Hoban et al. 2016; Sanford and Kelly 2011). To assess generality, future work will implement cross‐population common‐garden selection experiments, including multiple invasive‐ and native‐range sources, and analyze responses using mixed‐effects survival models (with population as a random effect) to quantify shared versus population‐specific components (Lotterhos and Whitlock 2015; McGaughran et al. 2024). Ultimately, incorporating the concept of evolutionary filtering at early invasion stages into risk assessment protocols and management frameworks holds great potential for enhancing our capacity to predict and prevent biological invasions. This integrative approach is particularly crucial given the accelerating pace of global environmental change, which is likely to alter invasion pressures and the adaptive landscapes faced by introduced species.

## Conflicts of Interest

The authors declare no conflicts of interest.

## Supporting information


**Data S1:** eva70177‐sup‐0001‐DataS1.docx.

## Data Availability

The data that support the findings of this study are openly available in NCBI GenBank at https://www.ncbi.nlm.nih.gov/, reference number PRJNA1315036.
