# Supplementary material for: Genomic Insights Into Early‐Stage Selective Filtering During the Transport Stage of Biological Invasions
Source: Evol Appl. 2025 Nov 6;18(11):e70177. doi: 10.1111/eva.70177 (PMC12592103; doi:10.1111/eva.70177)
Supplement: Supplementary file 1 — Data S1: eva70177‐sup‐0001‐DataS1.docx. [file EVA-18-e70177-s001.docx]

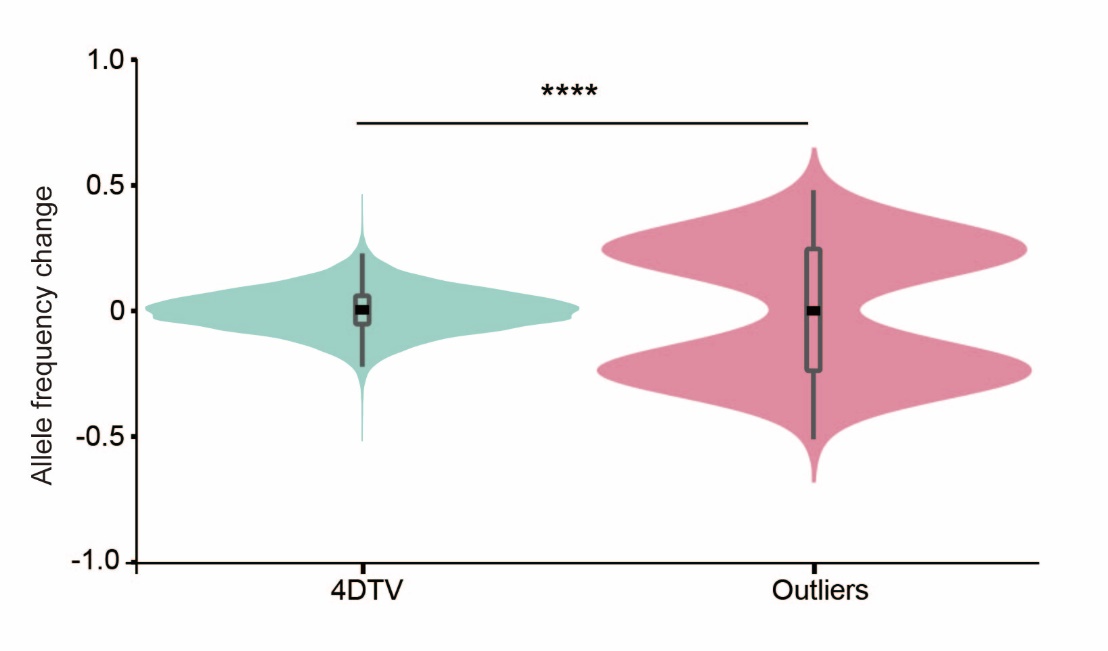


Figure S1 Violin plots comparing the distribution of allele frequency changes between 4DTV sites and outlier Single Nucleotide Polymorphisms (SNPs) within Composite Selection Score (CSS) regions. 4DTV sites are four-fold degenerate sites assumed to be neutral. **** indicates *p* < 0.0001 (two-sided Wilcoxon rank-sum test).


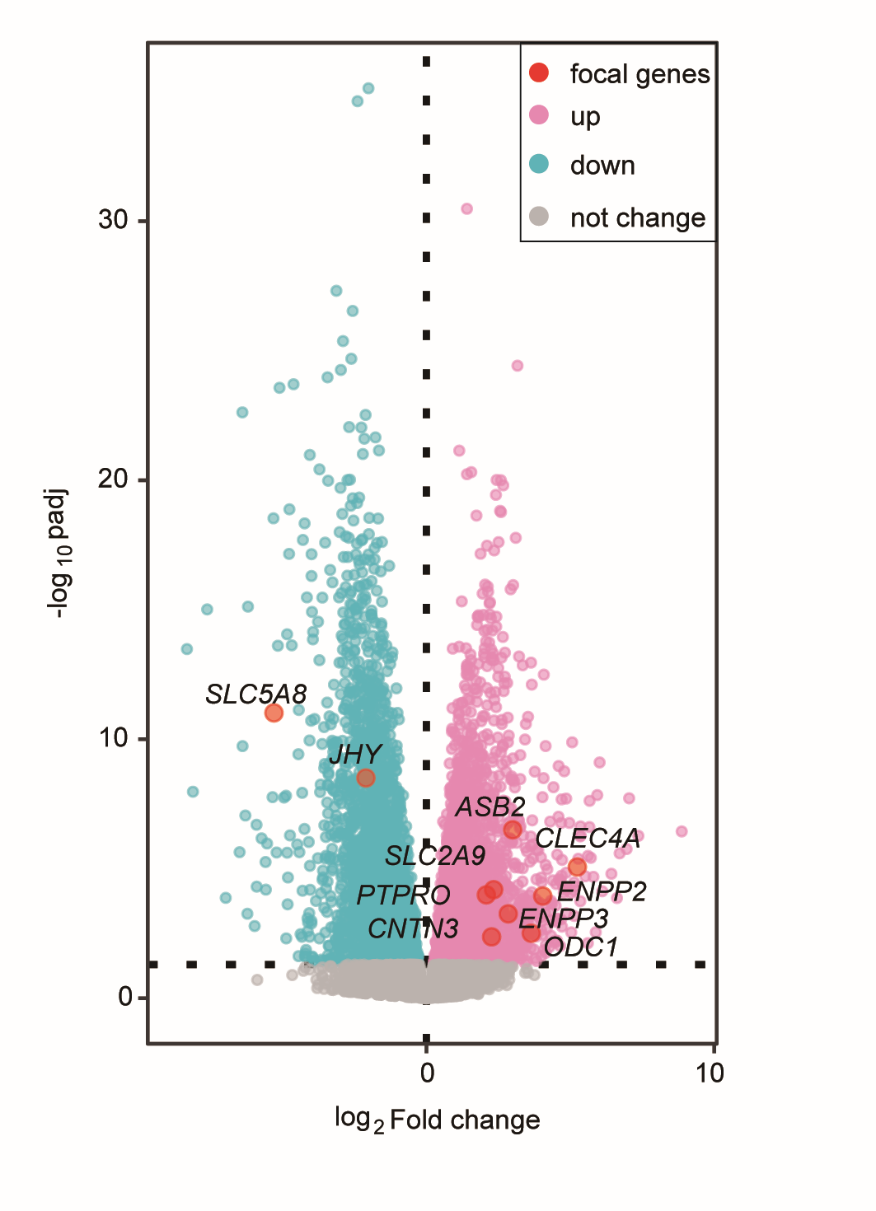


Figure S2 Volcano plot showing differential gene expression between treatment and control groups. Point colors indicate the direction of expression: pink for upregulated genes and blue for downregulated genes in the treatment group. Ten focal genes with significant differential expression and potential selection signatures are labeled and highlighted in red.

Table S1 Summary statistics of genetic diversity in control and treatment populations. *Ho*, observed heterozygosity; *He*, expected heterozygosity; *Pi*, nucleotide diversity; *Ar*, allelic richness.

| **Population** | ***Ho*** | ***He*** | ***Pi*** | ***Ar*** |
| --- | --- | --- | --- | --- |
| CR | 0.3769 | 0.3548 | 0.00302 | 1.3591 |
| HC1 | 0.3735 | 0.3545 | 0.00299 | 1.3584 |
| HC2 | 0.3736 | 0.3546 | 0.00286 | 1.3583 |
| HC3 | 0.3733 | 0.3544 | 0.00288 | 1.3589 |

**Table S2** List of genes associated with at least one focal Single nucleotide polymorphism (SNP) of putative functional relevance. SNPs are located in presumed promoter regions (upstream), downstream regions, untranslated regions (5' and 3' UTRs), or cause nonsynonymous amino acid substitutions.

| **GeneID** | **Start** | **End** | **Functional association** | **Gene symbol** | **upstream** | **downstream** | **3'UTR** | **5'UTR** | **missense** | **synonymous** |
| --- | --- | --- | --- | --- | --- | --- | --- | --- | --- | --- |
| KY21.Chr1.1238 | 8881620 | 8885736 | DNA binding | *NOBOX* | 0 | 0 | 0 | 0 | 0 | 0 |
| KY21.Chr1.1614 | 11162014 | 11168392 | Methyltransferase small domain | *N6AMT1* | 0 | 0 | 1 | 0 | 0 | 0 |
| KY21.Chr1.1615 | 11168951 | 11171775 | Protein ubiquitination | *FBXO9* | 0 | 1 | 0 | 0 | 0 | 0 |
| KY21.Chr1.1616 | 11172613 | 11175412 | Fc-gamma receptor I complex binding | *FGR* | 0 | 0 | 1 | 0 | 0 | 0 |
| KY21.Chr1.1617 | 11177508 | 11185705 | DNA damage repair and immune responses | *PARP9* | 0 | 0 | 0 | 0 | 0 | 1 |
| KY21.Chr1.1619 | 11196119 | 11200003 | alkaline phosphatase activity | *ALPL* | 1 | 0 | 0 | 0 | 0 | 1 |
| KY21.Chr1.1620 | 11200514 | 11210599 | peptidase inhibitor activity | *GLIPR2* | 1 | 0 | 0 | 0 | 0 | 0 |
| KY21.Chr1.1621 | 11206528 | 11210144 | peptidase inhibitor activity | *GLIPR2* | 0 | 1 | 0 | 0 | 0 | 0 |
| KY21.Chr1.1622 | 11213091 | 11220436 | ubiquitin-protein transferase activity | *MAGEL2* | 0 | 0 | 0 | 0 | 0 | 1 |
| KY21.Chr1.1891 | 12937486 | 12942551 | negative regulation of filopodium assembly | *CAPZB* | 0 | 0 | 0 | 0 | 0 | 0 |
| KY21.Chr1.2117 | 14572331 | 14596485 | nuclear pore complex assembly | *AHCTF1* | 0 | 0 | 0 | 0 | 0 | 0 |
| KY21.Chr1.495 | 3545568 | 3564389 | MAM domain, meprin/A5/mu | *MAMDC2* | 0 | 0 | 0 | 0 | 0 | 0 |
| KY21.Chr1.670 | 4759188 | 4813235 | Vacuolar protein | *VPS13C* | 0 | 0 | 0 | 0 | 0 | 0 |
| KY21.Chr1.913 | 6624994 | 6637456 | protein-cysteine S-acyltransferase activity | *ZDHHC8* | 0 | 0 | 0 | 0 | 0 | 1 |
| KY21.Chr10.23 | 186415 | 206844 | Belongs to the TRAFAC class myosin-kinesin ATPase superfamily. Myosin family | *MYO1E* | 0 | 0 | 0 | 0 | 0 | 3 |
| KY21.Chr10.686 | 4939604 | 4947491 | negative regulation of extracellular matrix disassembly | *FAP* | 0 | 0 | 0 | 0 | 0 | 0 |
| KY21.Chr10.687 | 4948926 | 4949554 | the degradation of glycogen | *GAA* | 1 | 0 | 0 | 0 | 0 | 0 |
| KY21.Chr11.308 | 1803575 | 1808675 | phospholipid metabolism | *CHKB* | 1 | 0 | 0 | 0 | 0 | 0 |
| KY21.Chr11.309 | 1809495 | 1813170 | small ribosomal subunit rRNA binding | *RPS13* | 1 | 0 | 0 | 0 | 0 | 0 |
| KY21.Chr11.310 | 1813166 | 1820636 | component of the small ribosomal subunit | *RPS13* | 0 | 1 | 0 | 0 | 0 | 2 |
| KY21.Chr11.311 | 1821115 | 1824014 | calcium ion binding | *AGRN* | 1 | 1 | 0 | 0 | 0 | 1 |
| KY21.Chr11.312 | 1824389 | 1826743 | cell surface receptor signaling pathway | *CD63* | 1 | 0 | 1 | 0 | 0 | 0 |
| KY21.Chr11.315 | 1836627 | 1861057 | negative chemotaxis | *SEMA4G* | 0 | 0 | 0 | 0 | 0 | 0 |
| KY21.Chr11.316 | 1848385 | 1863821 | microtubule cytoskeleton organization involved in establishment of planar polarity | *BRSK1* | 0 | 0 | 0 | 0 | 0 | 0 |
| KY21.Chr11.818 | 5857847 | 5861393 | potassium calcium-activated channel | *KCNN4* | 1 | 0 | 0 | 0 | 0 | 0 |
| KY21.Chr11.823 | 5881726 | 5896359 | RNA splicing, via transesterification reactions with bulged adenosine as nucleophile | *NOVA1* | 0 | 1 | 1 | 0 | 0 | 0 |
| KY21.Chr11.825 | 5893813 | 5894589 | regulate alternative splicing | *NOVA2* | 1 | 0 | 0 | 0 | 0 | 0 |
| KY21.Chr11.826 | 5896597 | 5899044 | Ectodermal ciliogenesis protein | *STUM* | 1 | 0 | 1 | 0 | 0 | 0 |
| KY21.Chr11.827 | 5900133 | 5903285 | Centriole, cilia and spindle-associated | *CCSAP* | 1 | 0 | 0 | 0 | 0 | 0 |
| KY21.Chr13.174 | 1480411 | 1504472 | mRNA 3'-UTR AU-rich region binding | *CPSF1* | 0 | 0 | 0 | 0 | 0 | 0 |
| KY21.Chr13.403 | 3660014 | 3688483 | integrin biosynthetic process | *COL11A1* | 0 | 0 | 0 | 0 | 0 | 0 |
| KY21.Chr13.450 | 4030362 | 4043891 | Modulator | *GPSM2* | 0 | 1 | 0 | 0 | 0 | 0 |
| KY21.Chr14.154 | 1218030 | 1229270 | Kynurenine alpha-aminoadipate aminotransferase | *AADAT* | 0 | 0 | 0 | 0 | 0 | 1 |
| KY21.Chr14.241 | 1853037 | 1913929 | Protocadherin Fat | *FAT1* | 0 | 0 | 0 | 0 | 0 | 0 |
| KY21.Chr14.253 | 1994430 | 2003364 | acts as an electrogenic sodium (Na(+)) and chloride (Cl-)-dependent sodium-coupled solute transporter | *SLC5A8* | 0 | 0 | 0 | 0 | 0 | 1 |
| KY21.Chr14.92 | 749434 | 778432 | negative regulation of protein autoubiquitination | *TAF1* | 0 | 0 | 0 | 0 | 0 | 0 |
| KY21.Chr2.1037 | 6194650 | 6198168 | protein-cysteine S-acyltransferase activity | *ZDHHC16* | 0 | 1 | 0 | 0 | 0 | 0 |
| KY21.Chr2.1038 | 6198382 | 6202021 | glycerone-phosphate O-acyltransferase activity | *GNPAT* | 1 | 0 | 0 | 0 | 0 | 0 |
| KY21.Chr2.1067 | 6411983 | 6426438 | protein serine/threonine kinase activity | *NEK10* | 0 | 0 | 0 | 0 | 0 | 0 |
| KY21.Chr2.1375 | 8433729 | 8437560 | Importin 9 | *IPO9* | 0 | 0 | 0 | 0 | 1 | 0 |
| KY21.Chr2.496 | 2826378 | 2840671 | positive regulation of microtubule nucleation | *DCTN1* | 0 | 1 | 0 | 0 | 0 | 0 |
| KY21.Chr2.52 | 320847 | 324345 | structural constituent of ribosome | *RPS3A* | 1 | 0 | 0 | 0 | 0 | 0 |
| KY21.Chr2.535 | 2978538 | 2980670 | zinc finger CCHC | *ZCCHC7* | 0 | 1 | 0 | 0 | 0 | 0 |
| KY21.Chr2.536 | 2980671 | 2982821 | methionyl-tRNA aminoacylation | *MARS2* | 0 | 0 | 0 | 0 | 0 | 0 |
| KY21.Chr2.645 | 3592204 | 3601731 | phosphodiesterase I activity | *ENPP2* | 0 | 0 | 0 | 0 | 0 | 0 |
| KY21.Chr2.646 | 3602918 | 3608092 | Hydrolase | *ENPP3* | 0 | 0 | 0 | 0 | 0 | 0 |
| KY21.Chr2.849 | 4930122 | 4937503 | mRNA cleavage | *CSTF3* | 0 | 0 | 0 | 0 | 0 | 0 |
| KY21.Chr2.850 | 4939202 | 4941870 | ornithine decarboxylase activity | *ODC1* | 0 | 0 | 0 | 0 | 0 | 1 |
| KY21.Chr2.937 | 5571190 | 5573720 | regulating intracellular protein transport | *USH2A* | 1 | 0 | 0 | 0 | 0 | 0 |
| KY21.Chr2.938 | 5574097 | 5578235 | Chromosome 2 open reading frame 81 | *C2orf81* | 1 | 0 | 0 | 0 | 1 | 0 |
| KY21.Chr2.941 | 5588513 | 5594499 | peptidyl-lysine oxidation | *LOXL4* | 0 | 1 | 0 | 0 | 1 | 0 |
| KY21.Chr3.10 | 52806 | 54975 | ubiquinol-cytochrome-c reductase activity | *UQCRFS1* | 0 | 1 | 0 | 0 | 0 | 0 |
| KY21.Chr3.319 | 1968965 | 1979091 | adhesion G protein-coupled receptor | *ADGRB1* | 0 | 0 | 0 | 0 | 1 | 0 |
| KY21.Chr3.374 | 2343426 | 2395329 | ATP-dependent microtubule motor activity, minus-end-directed | *DNAH17* | 0 | 0 | 0 | 0 | 0 | 0 |
| KY21.Chr3.41 | 211262 | 219823 | beta-endorphin binding | *IDE* | 0 | 1 | 0 | 0 | 0 | 0 |
| KY21.Chr3.42 | 212632 | 219990 | DNA methyltransferase | *DNMT3A* | 0 | 0 | 0 | 2 | 0 | 0 |
| KY21.Chr3.43 | 219871 | 221046 | positive regulation of androgen receptor activity | *bud31* | 1 | 1 | 1 | 0 | 0 | 0 |
| KY21.Chr3.44 | 221019 | 223024 | structural constituent of cuticle | *CDAN1* | 0 | 0 | 0 | 1 | 0 | 0 |
| KY21.Chr3.788 | 5386865 | 5389873 | intermembrane sphingolipid transfer activity | *COL4A3BP* | 0 | 0 | 0 | 0 | 0 | 1 |
| KY21.Chr3.9 | 50550 | 51770 | ciliary basal body organization | *CCP110* | 1 | 0 | 0 | 0 | 0 | 0 |
| KY21.Chr4.1000 | 6306664 | 6313503 | neural crest formation | *KLHL12* | 0 | 0 | 0 | 0 | 0 | 0 |
| KY21.Chr4.151 | 1246892 | 1257274 | positive regulation of oligodendrocyte differentiation | *PTPRO* | 0 | 1 | 0 | 0 | 0 | 0 |
| KY21.Chr4.233 | 1764461 | 1773873 | ribosomal small subunit binding | *PTCD3* | 0 | 0 | 0 | 0 | 0 | 1 |
| KY21.Chr4.463 | 3099511 | 3103979 | cytoplasmic translational initiation | *EIF3M* | 0 | 0 | 0 | 0 | 0 | 1 |
| KY21.Chr4.661 | 4111096 | 4144134 | synaptic vesicle exocytosis | *FER1L6* | 0 | 0 | 0 | 0 | 0 | 0 |
| KY21.Chr4.788 | 4936785 | 4943950 | diphthine-ammonia ligase activity | *DPH6* | 0 | 1 | 0 | 0 | 0 | 0 |
| KY21.Chr4.789 | 4943951 | 4944975 | tRNA wobble uridine modification | *ELP6* | 0 | 0 | 0 | 0 | 0 | 1 |
| KY21.Chr4.790 | 4944976 | 4946815 | DNA/RNA non-specific endonuclease | *ENDOD1* | 1 | 0 | 0 | 0 | 0 | 0 |
| KY21.Chr4.842 | 5245911 | 5251254 | negative regulation of store-operated calcium channel activity | *UBQLN1* | 1 | 0 | 0 | 0 | 0 | 1 |
| KY21.Chr4.843 | 5251746 | 5253623 | vesicle targeting, trans-Golgi to endosome | *WIPI2* | 0 | 0 | 1 | 0 | 0 | 0 |
| KY21.Chr4.846 | 5266723 | 5275867 | Cilium biogenesis/degradation | *JHY* | 0 | 0 | 0 | 0 | 0 | 0 |
| KY21.Chr4.999 | 6300494 | 6306560 | coreceptor activity involved in Wnt signaling pathway, planar cell polarity pathway | *Adamtsl1* | 0 | 1 | 0 | 0 | 0 | 0 |
| KY21.Chr5.367 | 2542770 | 2555030 | Dystroglycan 1 (Dystrophin-associated glycoprotein 1) | *DAG1* | 0 | 0 | 0 | 0 | 0 | 0 |
| KY21.Chr5.368 | 2556973 | 2585680 | protein ubiquitination | *RNF123* | 0 | 0 | 0 | 0 | 0 | 0 |
| KY21.Chr5.414 | 2908684 | 2914734 | phosphorylation of RNA polymerase II C-terminal domain | *GTF2H1* | 0 | 0 | 0 | 0 | 0 | 0 |
| KY21.Chr5.415 | 2915005 | 2921598 | Proposed core component of the chromatin remodeling INO80 complex which is involved in transcriptional regulation, DNA replication and probably DNA repair | *RUVBL2* | 0 | 0 | 0 | 0 | 0 | 0 |
| KY21.Chr5.442 | 3074443 | 3077105 | renal sodium ion absorption | *NDNL2* | 0 | 0 | 0 | 0 | 0 | 0 |
| KY21.Chr5.444 | 3079741 | 3082056 | peptide-O-fucosyltransferase activity | *POFUT1* | 0 | 0 | 1 | 0 | 0 | 0 |
| KY21.Chr5.445 | 3081925 | 3088610 | cell division | *SAC3D1* | 0 | 0 | 1 | 0 | 0 | 0 |
| KY21.Chr5.449 | 3097879 | 3100757 | carbohydrate binding | *CD207* | 0 | 1 | 0 | 0 | 1 | 0 |
| KY21.Chr5.450 | 3104312 | 3106444 | carbohydrate binding | *CLEC4A* | 0 | 0 | 1 | 0 | 0 | 0 |
| KY21.Chr6.173 | 1308639 | 1309426 | ATP hydrolysis activity | *LONP2* | 0 | 1 | 0 | 0 | 0 | 0 |
| KY21.Chr6.174 | 1310932 | 1311857 | ATP hydrolysis activity | *NLRP3* | 1 | 0 | 0 | 0 | 0 | 0 |
| KY21.Chr6.191 | 1415427 | 1420173 | transmembrane transporter activity | *SLC2A1* | 0 | 0 | 0 | 0 | 0 | 0 |
| KY21.Chr6.193 | 1429074 | 1434791 | transmembrane transporter activity | *SLC2A9* | 0 | 0 | 0 | 0 | 0 | 0 |
| KY21.Chr6.561 | 3832254 | 3838635 | prenylated protein catabolic process | *ZMPSTE24* | 0 | 0 | 0 | 0 | 0 | 0 |
| KY21.Chr6.562 | 3841552 | 3845229 | positive regulation of cortisol biosynthetic process | *WNT4* | 1 | 0 | 0 | 0 | 0 | 0 |
| KY21.Chr7.1138 | 8503712 | 8506972 | C2H2 zinc finger domain binding | *SRRM2* | 0 | 0 | 0 | 0 | 0 | 1 |
| KY21.Chr7.1144 | 8525909 | 8544088 | cholesterol binding | *OSBPL1A* | 0 | 0 | 0 | 0 | 0 | 0 |
| KY21.Chr7.1145 | 8544041 | 8549595 | optic cup structural organization | *TFAP2A* | 0 | 1 | 0 | 0 | 0 | 0 |
| KY21.Chr7.1146 | 8549940 | 8558729 | snRNA transcription by RNA polymerase III | *SNAPC4* | 0 | 1 | 0 | 0 | 0 | 1 |
| KY21.Chr7.1177 | 8752050 | 8757787 | SLU7 splicing factor homolog (S. cerevisiae) | *SLU7* | 0 | 1 | 0 | 0 | 0 | 1 |
| KY21.Chr7.1178 | 8757498 | 8762723 | cell motility in response to calcium ion | *SPAG16* | 0 | 0 | 0 | 0 | 0 | 0 |
| KY21.Chr7.1179 | 8762977 | 8767541 | protein ubiquitination | *ASB2* | 0 | 1 | 0 | 0 | 0 | 0 |
| KY21.Chr7.1181 | 8769794 | 8811610 | heavy chain 6 | *DNAH6* | 0 | 0 | 0 | 0 | 0 | 0 |
| KY21.Chr7.1185 | 8821461 | 8824944 | autophagy of peroxisome | *ACBD5* | 1 | 0 | 0 | 0 | 0 | 0 |
| KY21.Chr7.1186 | 8822519 | 8823567 | Transcription regulation | *RCOR1* | 0 | 1 | 0 | 0 | 0 | 0 |
| KY21.Chr7.1187 | 8825684 | 8831088 | Notch signaling pathway | *NOTCH1* | 1 | 0 | 0 | 0 | 0 | 0 |
| KY21.Chr7.469 | 3489027 | 3523751 | Nuclear pore complex scaffold, nucleoporins 186/192/205 | *NUP205* | 0 | 0 | 0 | 0 | 0 | 1 |
| KY21.Chr7.987 | 7405890 | 7410804 | negative regulation of apoptotic process | *CASP2* | 0 | 0 | 0 | 0 | 0 | 0 |
| KY21.Chr8.1114 | 6530321 | 6537203 | neurotrophin TRKA receptor binding | *GRB2* | 0 | 0 | 0 | 0 | 0 | 0 |
| KY21.Chr8.1243 | 7451938 | 7471034 | establishment of protein localization to juxtaparanode region of axon | *CNTN3* | 0 | 0 | 0 | 0 | 0 | 0 |
| KY21.Chr8.1244 | 7470980 | 7480899 | oxalate transmembrane transporter activity | *SLC26A5* | 0 | 0 | 0 | 0 | 0 | 0 |
| KY21.Chr8.752 | 4600080 | 4608430 | DOMON domain | *NOTCH3* | 0 | 1 | 0 | 0 | 0 | 0 |
| KY21.Chr8.753 | 4608843 | 4618003 | Belongs to the MCM family | *MCM6* | 0 | 1 | 0 | 0 | 0 | 0 |
| KY21.Chr9.113 | 728347 | 734712 | lens morphogenesis in camera-type eye | *TDRD7* | 0 | 0 | 0 | 0 | 0 | 1 |
| KY21.Chr9.1132 | 7921960 | 7939723 | ATPase activity, coupled to transmembrane movement of substances | *ABCA5* | 0 | 0 | 0 | 0 | 0 | 1 |
| KY21.Chr9.216 | 1511213 | 1518099 | Cell adhesion | *TNXB* | 0 | 0 | 0 | 0 | 0 | 1 |
| KY21.Chr9.291 | 1937353 | 1942664 | Lipid metabolism | *B3GALT2* | 0 | 0 | 0 | 0 | 0 | 0 |
| KY21.Chr9.310 | 2048928 | 2061858 | positive regulation of interleukin-4-mediated signaling pathway | *PARP14* | 0 | 0 | 0 | 0 | 0 | 1 |
| KY21.Chr9.312 | 2062496 | 2073420 | positive regulation of interleukin-4-mediated signaling pathway | *PARP14* | 0 | 0 | 0 | 0 | 0 | 0 |
| KY21.Chr9.316 | 2097995 | 2106944 | protein ubiquitination | *DTX3* | 0 | 0 | 0 | 0 | 1 | 0 |
| KY21.Chr9.748 | 5518497 | 5522852 | Cell adhesion | *TNXB* | 0 | 0 | 0 | 1 | 0 | 0 |
| KY21.Chr9.790 | 5765558 | 5772462 | very-long-chain-acyl-CoA dehydrogenase activity | *ACAD11* | 0 | 0 | 1 | 0 | 0 | 0 |
| KY21.Chr9.791 | 5772399 | 5775031 | endoribonuclease inhibitor activity | *TMBIM6* | 1 | 1 | 0 | 0 | 0 | 0 |
| KY21.Chr9.792 | 5776291 | 5794538 | Protein phosphatase 1 regulatory subunit 9A | *PPP1R9A* | 1 | 0 | 0 | 0 | 0 | 0 |

**Table S3** Gene Ontology (GO) enrichment analysis of candidate genes containing focal Single Nucleotide Polymorphisms (SNPs).

| Ontology | ID | Description | GeneRatio | *p.adjust* | geneID | Count |
| --- | --- | --- | --- | --- | --- | --- |
| BP | GO:0021682 | nerve maturation | 2/76 | 0.016 | KY21.Chr5.367.v1.SL1-1/KY21.Chr8.1243.v1.SL1-1 | 2 |
| BP | GO:0030505 | inorganic diphosphate transport | 2/76 | 0.016 | KY21.Chr2.645.v1.ND1-1/KY21.Chr2.646.v1.SL1-1 | 2 |
| BP | GO:0070667 | negative regulation of mast cell proliferation | 2/76 | 0.016 | KY21.Chr2.645.v1.ND1-1/KY21.Chr2.646.v1.SL1-1 | 2 |
| BP | GO:0001954 | positive regulation of cell-matrix adhesion | 4/76 | 0.028 | KY21.Chr2.645.v1.ND1-1/KY21.Chr2.646.v1.SL1-1/KY21.Chr5.367.v1.SL1-1/KY21.Chr6.562.v1.ND1-1 | 4 |
| BP | GO:0002276 | basophil activation involved in immune response | 2/76 | 0.035 | KY21.Chr2.645.v1.ND1-1/KY21.Chr2.646.v1.SL1-1 | 2 |
| BP | GO:0045575 | basophil activation | 2/76 | 0.035 | KY21.Chr2.645.v1.ND1-1/KY21.Chr2.646.v1.SL1-1 | 2 |
| BP | GO:0055062 | phosphate ion homeostasis | 3/76 | 0.035 | KY21.Chr2.645.v1.ND1-1/KY21.Chr2.646.v1.SL1-1/KY21.Chr7.1145.v3.ND2-1 | 3 |
| BP | GO:0072505 | divalent inorganic anion homeostasis | 3/76 | 0.035 | KY21.Chr2.645.v1.ND1-1/KY21.Chr2.646.v1.SL1-1/KY21.Chr7.1145.v3.ND2-1 | 3 |
| BP | GO:0072506 | trivalent inorganic anion homeostasis | 3/76 | 0.035 | KY21.Chr2.645.v1.ND1-1/KY21.Chr2.646.v1.SL1-1/KY21.Chr7.1145.v3.ND2-1 | 3 |
| BP | GO:0006771 | riboflavin metabolic process | 2/76 | 0.035 | KY21.Chr2.645.v1.ND1-1/KY21.Chr2.646.v1.SL1-1 | 2 |
| BP | GO:0034638 | phosphatidylcholine catabolic process | 2/76 | 0.035 | KY21.Chr2.645.v1.ND1-1/KY21.Chr2.646.v1.SL1-1 | 2 |
| BP | GO:0061744 | motor behavior | 2/76 | 0.035 | KY21.Chr10.686.v1.SL1-1/KY21.Chr2.496.v1.SL1-1 | 2 |
| BP | GO:0042726 | flavin-containing compound metabolic process | 2/76 | 0.035 | KY21.Chr2.645.v1.ND1-1/KY21.Chr2.646.v1.SL1-1 | 2 |
| BP | GO:0070662 | mast cell proliferation | 2/76 | 0.035 | KY21.Chr2.645.v1.ND1-1/KY21.Chr2.646.v1.SL1-1 | 2 |
| BP | GO:0070666 | regulation of mast cell proliferation | 2/76 | 0.035 | KY21.Chr2.645.v1.ND1-1/KY21.Chr2.646.v1.SL1-1 | 2 |
| BP | GO:1902216 | positive regulation of interleukin-4-mediated signaling pathway | 2/76 | 0.039 | KY21.Chr9.310.v1.ND1-1/KY21.Chr9.312.v1.SL1-1 | 2 |
| BP | GO:0033006 | regulation of mast cell activation involved in immune response | 3/76 | 0.040 | KY21.Chr1.1616.v1.ND1-1/KY21.Chr2.645.v1.ND1-1/KY21.Chr2.646.v1.SL1-1 | 3 |
| BP | GO:0051894 | positive regulation of focal adhesion assembly | 3/76 | 0.040 | KY21.Chr2.645.v1.ND1-1/KY21.Chr2.646.v1.SL1-1/KY21.Chr6.562.v1.ND1-1 | 3 |
| BP | GO:0150117 | positive regulation of cell-substrate junction organization | 3/76 | 0.040 | KY21.Chr2.645.v1.ND1-1/KY21.Chr2.646.v1.SL1-1/KY21.Chr6.562.v1.ND1-1 | 3 |
| BP | GO:1902214 | regulation of interleukin-4-mediated signaling pathway | 2/76 | 0.040 | KY21.Chr9.310.v1.ND1-1/KY21.Chr9.312.v1.SL1-1 | 2 |
| BP | GO:0050731 | positive regulation of peptidyl-tyrosine phosphorylation | 5/76 | 0.040 | KY21.Chr2.645.v1.ND1-1/KY21.Chr2.646.v1.SL1-1/KY21.Chr8.1243.v1.SL1-1/KY21.Chr9.310.v1.ND1-1/KY21.Chr9.312.v1.SL1-1 | 5 |
| BP | GO:0050777 | negative regulation of immune response | 5/76 | 0.040 | KY21.Chr2.645.v1.ND1-1/KY21.Chr2.646.v1.SL1-1/KY21.Chr2.941.v1.ND1-1/KY21.Chr9.310.v1.ND1-1/KY21.Chr9.312.v1.SL1-1 | 5 |
| BP | GO:0002279 | mast cell activation involved in immune response | 3/76 | 0.040 | KY21.Chr1.1616.v1.ND1-1/KY21.Chr2.645.v1.ND1-1/KY21.Chr2.646.v1.SL1-1 | 3 |
| BP | GO:0033007 | negative regulation of mast cell activation involved in immune response | 2/76 | 0.040 | KY21.Chr2.645.v1.ND1-1/KY21.Chr2.646.v1.SL1-1 | 2 |
| BP | GO:1903393 | positive regulation of adherens junction organization | 3/76 | 0.047 | KY21.Chr2.645.v1.ND1-1/KY21.Chr2.646.v1.SL1-1/KY21.Chr6.562.v1.ND1-1 | 3 |
| BP | GO:0033004 | negative regulation of mast cell activation | 2/76 | 0.047 | KY21.Chr2.645.v1.ND1-1/KY21.Chr2.646.v1.SL1-1 | 2 |
| MF | GO:0004528 | phosphodiesterase I activity | 2/69 | 0.020 | KY21.Chr2.645.v1.ND1-1/KY21.Chr2.646.v1.SL1-1 | 2 |
| MF | GO:0001046 | core promoter sequence-specific DNA binding | 4/69 | 0.025 | KY21.Chr14.92.v1.SL1-1/KY21.Chr3.41.v1.nonSL1-1/KY21.Chr5.415.v1.SL1-1/KY21.Chr7.1145.v3.ND2-1 | 4 |
| MF | GO:0000979 | RNA polymerase II core promoter sequence-specific DNA binding | 3/69 | 0.025 | KY21.Chr14.92.v1.SL1-1/KY21.Chr5.415.v1.SL1-1/KY21.Chr7.1145.v3.ND2-1 | 3 |
| MF | GO:0004551 | dinucleotide phosphatase activity | 2/69 | 0.025 | KY21.Chr2.645.v1.ND1-1/KY21.Chr2.646.v1.SL1-1 | 2 |
| MF | GO:0031749 | D2 dopamine receptor binding | 2/69 | 0.025 | KY21.Chr11.312.v1.SL1-1/KY21.Chr9.792.v2.SL1-1 | 2 |
| MF | GO:0047391 | alkylglycerophosphoethanolamine phosphodiesterase activity | 2/69 | 0.025 | KY21.Chr2.645.v1.ND1-1/KY21.Chr2.646.v1.SL1-1 | 2 |
| MF | GO:0050656 | 3'-phosphoadenosine 5'-phosphosulfate binding | 2/69 | 0.025 | KY21.Chr2.645.v1.ND1-1/KY21.Chr2.646.v1.SL1-1 | 2 |
